# Measuring couple relationship quality in a rural African population: Validation of a Couple Functionality Assessment Tool in Malawi

**DOI:** 10.1371/journal.pone.0188561

**Published:** 2017-11-30

**Authors:** Allison Ruark, Rachel Chase, John Hembling, Valerie Rhoe Davis, Paul Clayton Perrin, Dorothy Brewster-Lee

**Affiliations:** 1 Department of Medicine, Brown University, Providence, Rhode Island, United States of America; 2 Department of International Health, Johns Hopkins University School of Public Health, Baltimore, Maryland, United States of America; 3 Catholic Relief Services, Baltimore, Maryland, United States of America; Yokohama City University, JAPAN

## Abstract

Available data suggest that individual and family well-being are linked to the quality of women’s and men’s couple relationships, but few tools exist to assess couple relationship functioning in low- and middle-income countries. In response to this gap, Catholic Relief Services has developed a Couple Functionality Assessment Tool (CFAT) to capture valid and reliable data on various domains of relationship quality. This tool is designed to be used by interventions which aim to improve couple and family well-being as a means of measuring the effectiveness of these interventions, particularly related to couple relationship quality. We carried out a validation study of the CFAT among 401 married and cohabiting adults (203 women and 198 men) in rural Chikhwawa District, Malawi. Using psychometric scales, the CFAT addressed six domains of couple relationship quality (intimacy, partner support, sexual satisfaction, gender roles, decision-making, and communication and conflict management), and included questions on intimate partner violence. We used exploratory factor analysis to assess scale performance of each domain and produce a shortened Relationship Quality Index (RQI) composed of items from five relationship quality domains. This article reports the performance of the RQI. Internal reliability and validity of the RQI were found to be good. Regression analyses examined the relationship of the RQI to outcomes important to health and development: intra-household cooperation, positive health behaviors, intimate partner violence, and gender-equitable norms. We found many significant correlations between RQI scores and these couple- and family-level development issues. There is a need to further validate the tool with use in other populations as well as to continue to explore whether the observed linkages between couple functionality and development outcomes are causal relationships.

## Introduction

The quality of women’s and men’s couple relationships has implications for their health and well-being as well as the health and well-being of their families. Whereas decades of research in high-income countries have shown that marital quality impacts physical and emotional health [1] and child well-being [2], research has only recently begun to investigate associations between health and the quality of couple relationships in low- and middle-income countries (LMIC). Relationship quality has been defined in a variety of ways in the literature. Lawrence and colleagues, in a review of research across multiple disciplines, identified the following five dimensions of relationship quality: emotional intimacy; quality of the sexual relationship; inter-partner support; ability to share power in the relationship; and communication and conflict management [3,4]. All research reviewed by Lawrence and colleagues appears to be from the United States, and we could not identify in the literature a definition of relationship quality based on research from LMIC.

Research in African contexts has linked relationship quality to uptake and experience of HIV testing and counseling in Tanzania [5] and South Africa [6] and risk of HIV infection in Uganda [7]. Cox and colleagues used psychometric scales measuring commitment, trust, and communication to assess Ghanaian couples and found high reported relationship quality for men and women, and that relationship satisfaction was correlated with use and type of contraception [8]. In Kenya, Kwena and colleagues assessed sexual satisfaction among married couples in fishing communities, and found that sexual concurrency was more likely among couples who reported low marital sexual satisfaction (specifically, men being denied sex) [9]. In South Africa, three innovative interventions have attempted to enhance couple relationship quality in order to reduce HIV risk [10–12], and have documented linkages between relationship quality and outcomes such as shared power [10] and decreased intimate partner violence (IPV) [11]. IPV, a sign of poor relationship quality, carries with it many risks, including increased risk of HIV infection [13,14].

Couple relationship quality has also been linked to the health and well-being of children in the household, beginning with prenatal care. In South Africa, poor communication within the couple was found to impede male partner involvement in prevention of mother-to-child transmission of HIV [15]. In India, women with better marital relationships were more likely to utilize maternal health care services [16] and mothers with greater participation in household decision-making had healthier infants [17]. Positive spousal communication has been linked to exclusive breastfeeding in Lao PDR [18] and use by children of insecticide-treated bed nets in Nigeria [19].

The Couple Functionality Assessment Tool (CFAT) was developed by Catholic Relief Services (CRS) to be used by development programs globally to assess various domains of couple relationship quality and functionality. We designed the CFAT to assist in the design and evaluation of programs that have an explicit aim of enhancing aspects of couple functionality to prevent IPV and improve outcomes in early childhood development, nutrition, household economic strengthening, and agriculture. The CFAT is one of the first such tools to be developed for assessing relationship quality across multiple domains for use in LMIC. In this article we report on findings from the validation study conducted in rural Malawi. Specifically, we report the development and performance of the subset of CFAT items that comprise the Relationship Quality Index (RQI), including the linkages between the RQI and intra-household cooperation, positive health behaviors, IPV, and gender-equitable norms.

## Methods

### Study procedures

We carried out this study in rural Chikhwawa District, Malawi. Chikhwawa District, located in southern Malawi along the Mozambique border, has a population of approximately 350,000. As part of an ongoing food security project in Chikhwawa District, CRS systematically compiled household lists for sub-district areas known as Traditional Authorities (TAs) and smaller Group Village Head (GVH) areas. We selected a random sample of these subdistricts from those TAs and GVHs for which household lists were available and which were reachable by land, without requiring travel by boat. We systematically sampled participants from these household lists.

Participants were eligible if they were married or cohabiting, had been in the relationship one year or more, were currently living with their partner and the partner was available to be interviewed, and the female partner was between the ages of 20 and 39. We systematically allocated couples to one of three groups: both partners interviewed, woman only interviewed, or man only interviewed. This allowed us to investigate whether there was an effect from knowing one’s partner was also being interviewed.

We translated the CFAT into Chichewa, pilot tested it, and addressed translation issues with the help of Chichewa-speaking research assistants before beginning data collection. Trained same-gender data collectors conducted interviews in Chichewa and obtained signed informed consent prior to the interview. All interviews were conducted at or near participants’ homes in a place where privacy could be ensured, and couples were interviewed separately.

The National Health Sciences Research Committee (NHSRC) of Malawi approved the study (Protocol 15/7/1445).

### Measures

#### Independent variables (relationship quality domains)

We selected six domains of relationship quality for inclusion in the CFAT based on a review of the literature by Lawrence and colleagues [3,4] and key informant interviews with CRS technical experts: intimacy, partner support, sexual satisfaction, constructive communication, gender roles, and decision-making. We identified measures for these domains through the suggestions of experts, literature searches, and review of all scales described in the comprehensive *Handbook of Family Measurement Techniques* [20]. The majority of measures reviewed had been developed for use in the United States or other high-income countries, and for most, there was no evidence of their use in LMIC.

We selected a scale or set of questions to measure each domain in consultation with CRS technical experts and based on several factors. First, we considered the cross-cultural transferability of measures and whether they would be easily understood and relevant in a wide variety of cultures. Second, when possible we used measures that had been used previously and performed well in LMIC. For measures for which we could only find evidence of use in the United States or other high-income countries, we prioritized measures which had been widely used in published research over those which had been less widely used. Consultation with CRS technical experts about the choice of measures also served to assess face and content validity of the measures. In one case, we made a change in wording in order to make a measure more cross-culturally applicable.

*Intimacy* was measured using the 12-item Intimacy Subscale of Sternberg’s Triangular Love Scale [21], which asks participants to indicate agreement with statements such as, “I have a warm and comfortable relationship with my partner.” Responses were scored on a Likert scale ranging from 1 (not at all) to 5 (very much).

*Partner support* was measured using an 8-item subscale from the Husband’s and Wives’ Emotion Work Scale [22], which asks participants how often they both give and receive the following types of support from their partners: “let my partner know I have faith in him/her,” “offer encouragement to my partner,” “stick by my partner in times of trouble,” and “offer advice when my partner is faced with a problem.” Responses were scored on a Likert scale ranging from 1 (never) to 5 (always).

*Sexual satisfaction* was measured using the 25-item Index of Sexual Satisfaction (ISS) [23], which asks participants about their level of agreement with various measures of sexual satisfaction. Thirteen items reflected a lack of satisfaction and were reverse-scored. At the recommendation of CRS technical experts, the item “Sex is *fun* for my partner and me” was re-worded as “Sex is *satisfying* for my partner and me.” Responses were scored on a Likert scale ranging from 1 (none of the time) to 5 (all of the time).

*Constructive communication* was measured using two subscales from the Communication Patterns Questionnaire [24]. A 3-item constructive subscale asks participants whether they practice positive communication patterns such as trying to discuss the problem. A 4-item, reverse-scored destructive communication subscale asks participants whether they practice negative communication patterns such as blaming, accusing, and criticizing each other. Responses were scored on a Likert scale ranging from 1 (very unlikely) to 5 (very likely).

*Gender roles* were measured using the Domestic Chores and Daily Life Domain subscale of the Gender Equitable Men (GEM) Scale [25,26], with the questions adapted to address a couple’s relationship, rather than broader social norms. For example, the statement, “Changing diapers, giving a bath, and feeding kids is the mother’s responsibility” was adapted to (for women): “In my household, changing diapers, giving a bath, and feeding kids is my responsibility and not my husband’s/partner’s.” Responses were scored on a 3-item Likert scale: 1 (do not agree), 2 (partially agree), and 3 (agree).

*Decision-making* was measured using 6 questions about household decision-making from the latest Malawi Demographic and Health Survey (DHS) [27]. Participants were asked who usually makes decisions regarding matters such as health care or household purchases (wife, husband, wife and husband jointly, or someone else). Participants were coded according to the proportion of decisions for which they reported sole decision-making power, joint decision-making power (with spouse or partner), or no decision-making power.

The RQI was developed as an index of these domains, with each domain shortened into a high-performing scale via exploratory factor analysis. For this analysis, participants were divided into four groups: married women, married men, women who were unmarried but cohabiting, and men who were unmarried but cohabiting. Items were omitted if they had very low absolute factor loadings (<0.30) for any group, low absolute factor loadings (<0.04) for multiple groups, or high absolute factor loadings in different directions when comparing two groups (for example, 0.54 in one group and -0.60 in another group). Items frequently performed less well among unmarried but cohabiting men compared to the other three groups. Iterative factor analysis and item omission aimed to improve the performance of each scale in all four groups and established internal construct validity of the scales. In all cases, only one factor was retained for each scale as in no scale did all groups indicate a two-factor solution. Cronbach’s alpha was used to compare the reliability of the original versus shortened scales. Unreported assessments conducted during factor analysis include eigenvalues, Bartlett’s test of sphericity, and the Kaiser—Meyer—Olkin test of sampling adequacy.

#### Dependent variables (outcomes)

Three sets of behaviors were treated as dependent variables (outcomes of interest) in this study: intra-household cooperation (hereafter referred to as “household cooperation”), positive health behaviors, and IPV. In addition, gender-equitable norms were assessed with a single item. These variables were identified based on a literature review (particularly, evidence that a behavior was associated with a construct of relationship quality, or relationship quality generally) and through key informant interviews with CRS technical experts. Correlating relationship quality to these outcomes of interest allowed us to establish criterion validity of the relationship quality measures.

*Household cooperation* was assessed using the following four items, which were developed by CRS technical experts based on their knowledge of important aspects of household cooperation: “My partner and I have decided together how many children we want to have,” “My partner and I decide together how to manage our household budget,” “My partner and I have a financial plan to which we both contribute our incomes,” and “My partner and I have talked together about what to do at times when there wasn’t enough food in the household.” All items were ranked on a Likert scale ranging from 1 (strongly disagree) to 5 (strongly agree). For analysis, these items were treated as binary, with responses of “agree” or “strongly agree” being coded as practicing the behavior.

*Health behaviors* were assessed by asking women who had been pregnant in the last 12 months the number of times they had received antenatal care, using a question from the DHS [27]. In addition, we added a question regarding whether a woman’s partner had attended at least one antenatal care visit with her. Men and women were also asked if they had been tested for HIV and shared their HIV status with each other, which was treated as a binary variable (1 if the participant reported that both partners had been tested and shared their status, 0 if this was not the case).

*IPV* was assessed among female participants using the entire IPV module from the DHS [27]. This module contains 24 questions that address a partner’s controlling behavior, emotional violence, and physical and sexual violence. Male participants were also asked if their female partners had ever inflicted emotional or physical violence. Both women and men were asked if they had ever perpetrated physical violence against a partner. For each type of violence, a participant was coded as experiencing violence if he or she reported ever experiencing one or more instances of that type of violence. Questions about controlling behavior referred to present experience with the current partner.

*Gender-equitable norms* were assessed using the single item “My partner deserves the best or largest portion of food at mealtimes” (for women), and “I deserve the best or largest portion of food at mealtimes” (for men). Responses were ranked on a Likert scale ranging from 1 (strongly disagree) to 5 (strongly agree) but for purposes of analysis were made binary, with responses of “agree” or “strongly agree” being coded as agreement with the statement.

### Statistical analysis

All variables were assessed by gender for descriptive purposes and comparison using chi-squared statistics for categorical variables and t-tests for ordinal variables. We assessed the predictive value on each outcome variable of each relationship quality domain using logistic regressions, adjusted for age, relationship duration, number of children in household, age difference between partners (man <5 years older, 5–9 years older, or 10+ years older), participant education level (no education, primary only, secondary or higher education), and marital status (married or living with partner). We selected these covariates based on a review of the literature, and assessed women and men separately.

In the five domains that constituted psychometric scales (intimacy, partner support, constructive communication, sexual satisfaction, and gender roles), we summed the scores of all individual items within each scale. We scored the decision-making domain according to what proportion of decisions were made jointly. We assessed collinearity for each independent variable; in no case did a variance inflation factor (VIF) exceed 4, indicating that collinearity was acceptably low. Finally, we calculated a RQI score by giving equal weight to the domains of relationship quality retained in the final measurement model. Using the regression models, we also calculated predicted probabilities of each dependent variable (outcome of interest) for women and men at 25-point intervals of the relationship quality score. We performed all analyses using STATA 13.1.

## Results

### Descriptive characteristics

We administered the CFAT to 203 women and 198 men, including 89 couples and 223 individuals whose partners were not interviewed. Table 1 gives demographic characteristics of the sample. Women were between the ages of 20 and 39 (mean age 28.5), while men were between the ages of 20 and 57 (mean age 33.6). Most participants were Protestant Christian (85%), married (68%), and had primary-level education (69%). Nearly half (44%) of participants were from the Manganja sociolinguistic group, 39% were Sena, and the rest were from other smaller groups. Men were significantly older and better educated than women, and also more likely to report that they were married (versus cohabiting) and less likely to report that they were polygamous.

**Table 1:** Characteristics of participants in Malawi CFAT validation study.

	Women (n = 203)		Men (n = 198)		Total (n = 401)	
	%	mean (SD)	%	mean (SD)	%	mean (SD)
Age (years)*		28.5 (5.3)		33.6 (6.6)		31.0 (6.5)
Duration of marriage/partnership (years)		9.9 (5.4)		9.6 (5.9)		9.8 (5.7)
Age difference between partners (years)*		5.4 (3.8)		6.4 (4.5)		5.9 (4.2)
Marital status*						
Married	61		76		68	
Cohabiting	39		24		32	
Polygamous marriage*^†^	13		4		8	
Education*						
No formal education	14		7		10	
Primary	78		60		69	
Secondary	8		32		20	
Tertiary	<1		1		1	
Ethnic group						
Manganja	46		43		44	
Sena	40		38		39	
Lomwe	8		11		9	
Other	6		9		7	
Religion						
Catholic	10		11		11	
Protestant	89		81		85	
Muslim	1		2		2	
No religion	0		6		3	

* Gender difference significant at p < .05 (using t-tests for continuous variables and chi-squared statistics for categorical variables).

^**†**^ Answering “Yes” to the question, “Do you have more than one wife or woman you live with as if married?” (men), or “Does your husband/partner have other wives or does he live with other women as if married?” (women).

Most women and men reported cooperative behaviors in the household, although women were significantly more likely than men to report that they had a joint financial plan with their partners (Table 2). Women generally agreed that men deserved the best or largest portion of food at mealtimes (75% of married and cohabiting women) while only a minority of men agreed (17% of married men and 9% of cohabiting men). The great majority of participants reported that they and their partners had been tested for HIV and shared their HIV status with each other (86% of married women, 99% of cohabiting women, 84% of married men, 91% of cohabiting men). For cohabitors, the difference between men and women was significant. Around half of women said that they had attended 4 or more antenatal visits at their last (or current) pregnancy (50% of married women, 45% of cohabiting women), while a higher proportion said their partner had attended at least one visit with them (54% of married women, 68% of cohabiting women).

**Table 2:** Dependent variables (outcomes).

	Married women (n = 123)	Cohabiting women (n = 80)	Married men (n = 151)	Cohabiting men (n = 47)
	%	%	%	%
*Household cooperation—decided with partner on*				
Number of children	72	85	77	72
Household budget	79	94	81	85
Joint financial plan	72	83	59*	60*
Plan during food scarcity	89	98	91	91
*Gender norms*				
Man deserves best/largest portion of food	75	75	17*	9*
*Health behaviors*				
Attended 4 or more ante-natal visits at last/ current pregnancy^†^	50	45	–	–
Partner attended at least one ante-natal visit at last/current pregnancy^†^	54	68	–	–
Both partners have been tested for HIV and mutually shared status	86	99	84	91*
*Experience of intimate partner violence*				
Controlling behavior by partner	59	63	–	–
Emotional violence by partner, ever	31	34	17*	26
Physical violence by partner, ever	26	31	–	–
Sexual violence by partner, ever	7	3	–	–
Perpetrated physical violence against partner, ever	2	5	25*	40*

^**†**^ Among all women who had ever been pregnant (119 married women, 78 cohabiting women).

* Gender difference significant at p < .05 for individuals with same marital status (using chi-squared statistics).

A majority of women reported ever having experienced one or more types of controlling behavior by their current partners (59% of married women and 63% of cohabiting women). Fewer women reported ever experiencing emotional violence (31% of married women and 34% of cohabiting women), physical violence (26% of married women and 31% of cohabiting women), or sexual violence (7% of married women and 3% of cohabiting women) from partners. Men were less likely than women to say they had experienced emotional violence from a partner (17% of married men and 26% of cohabiting men), with the difference between married women and men being significant. Only a few women reported ever having perpetrated violence against their partners (2% of married women and 5% of cohabiting women), whereas higher rates of men reported having perpetrated violence against partners (25% of married men and 40% of cohabiting men). The differences by gender were statistically significant for both married and cohabiting individuals.

### Final measurement model

Based on factor loadings and predictive ability, we reduced the total number of items in the RQI from 56 to 22 (plus the 6 items measuring decision-making, all of which were retained) and dropped the gender roles scale entirely due to poor performance of the scale. We standardized the scores for the remaining five relationship quality domains to have a minimum value of 0 (indicating low relationship quality) and a maximum value of 100 (indicating high relationship quality). The final 28-item measurement model showed high internal consistency (Cronbach’s alpha of 0.88).

#### Intimacy

We retained five items. Cronbach’s alpha was 0.86 for both the 5-item and 12-item scales.

#### Partner support

We retained four items. Cronbach’s alpha for the 4-item scale was 0.82 compared to alpha of 0.85 for the 8-item scale.

#### Sexual satisfaction

We retained six items. Cronbach’s alpha for the 6-item scale was 0.82 compared to alpha of 0.79 for the 25-item scale.

#### Constructive communication

The three items in the constructive communication subscale did not load onto a factor with the four items of the destructive communication subscale, or with each other. The 4-item destructive communication subscale did perform well as a scale in factor analysis. Cronbach’s alpha of the entire 7-item scale was 0.60, while alpha of the 4-item destructive communication scale was 0.75. As all seven items showed significant associations with the dependent variables, we retained them all in the final measurement model.

#### Gender roles

This scale performed poorly amongst the four gender and marital status categories (with 6 of 20 factor loadings across the four groups being < .40) and had relatively low internal consistency (Cronbach’s alpha of 0.69). The gender roles scale also showed poor predictive validity. For women, the only significant associations with dependent variables were negative. Women who reported more equitable gender roles were more likely to report experiencing physical violence from their partners and perpetrating physical violence against their partners (p < .05 in multivariate analysis for both associations). For men, more equitable gender roles were positively associated with two dependent variables. Men with more equitable gender roles were less likely say they deserved the best or largest portion of food at mealtimes (p < .01) and less likely to have perpetrated physical violence against partners (p < .05). Based on the poor performance of the scale in factor analysis and the mixed findings regarding associations with dependent variables, the gender roles scale was removed from the final measurement model.

#### Decision-making

As these questions did not constitute a scale, they were not evaluated using exploratory factor analysis and Cronbach’s alpha. All six questions were retained and were used to generate two different measurements: proportion of decisions which were made jointly by both partners, and proportion of decisions which were made solely by the participant. Joint decision-making was positively associated with multiple dependent variables for both men and women. However, sole decision-making by the participant was negatively associated with multiple dependent variables, and never positively associated with a dependent variable. Men and women who reported making a greater proportion of decisions alone were significantly less likely to report household cooperation and positive health behaviors, significantly more likely to report perpetrating physical violence against a partner, and men were significantly more likely to report emotional violence by a partner. Therefore joint decision-making was included as a domain of relationship quality in the final measurement model, with a score of 0 indicating no decisions made jointly, and a score of 100 indicating all decisions made jointly.

#### Relationship Quality Index (RQI)

The RQI was calculated as a composite relationship quality score of 0 to 100 points by equally weighting the following five domains, with each domain contributing 20 points: intimacy (5 items), partner support (4 items), sexual satisfaction (6 items), constructive communication (7 items), and joint decision-making (6 items).

Participants generally rated their relationship quality as high, with mean scores in each relationship quality domain generally exceeding 75 out of 100 (Table 3). Married women reported the lowest mean scores in 3 of 5 domains. The lowest scores were in the decision-making domain, with the mean number of decisions made jointly 50% or below for all groups except married men, and cohabiting men reporting a mean of only 30% of decisions made jointly. In addition, a full quarter of cohabiting women (20 of 80) and nearly a third of cohabiting men (15 of 47) reported that they made no decisions jointly with their partners. Married men scored significantly higher than married women on each relationship quality domain. Among cohabitors, there was a significant gender difference only regarding joint decision-making, with women reporting a higher proportion of decisions made jointly.

**Table 3:** Independent variables (relationship quality domains), final measurement model.

	Married women (n = 123)	Cohabiting women (n = 80)	Married men (n = 151)	Cohabiting men (n = 47)
	mean (SD)	mean (SD)	mean (SD)	mean (SD)
Intimacy (5 items)	84.0 (19.9)	86.4 (16.0)	88.5 (12.3)*	82.7 (12.5)
Partner support (4 items)	69.5 (28.3)	77.3 (21.7)	76.9 (20.4)*	74.3 (16.8)
Sexual satisfaction (6 items)	81.6 (18.4)	84.3 (14.4)	87.6 (13.8)*	82.5 (11.7)
Constructive communication (7 items)	77.3 (13.3)	79.1 (13.8)	82.8 (12.8)*	83.5 (11.1)
Joint decision-making^†^ (6 items)	40.6 (31.8)	42.3 (33.4)	52.6 (34.3)*	30.4 (28.2)*
Relationship Quality Index (RQI) (28 items)	70.6 (16.1)	73.9 (13.1)	77.4 (13.2)*	70.3 (9.3)

*Note*: All variables standardized to scale of 0 to 100, in which higher scores denote a higher-quality or more gender-equitable relationship.

* Gender difference significant at p < .05 for individuals with same marital status.

^**†**^ Proportion of total household decisions made jointly with partner according to respondent’s report, on scale of 0 to 100.

### Association of outcomes to relationship quality domains

**Fig 1:**
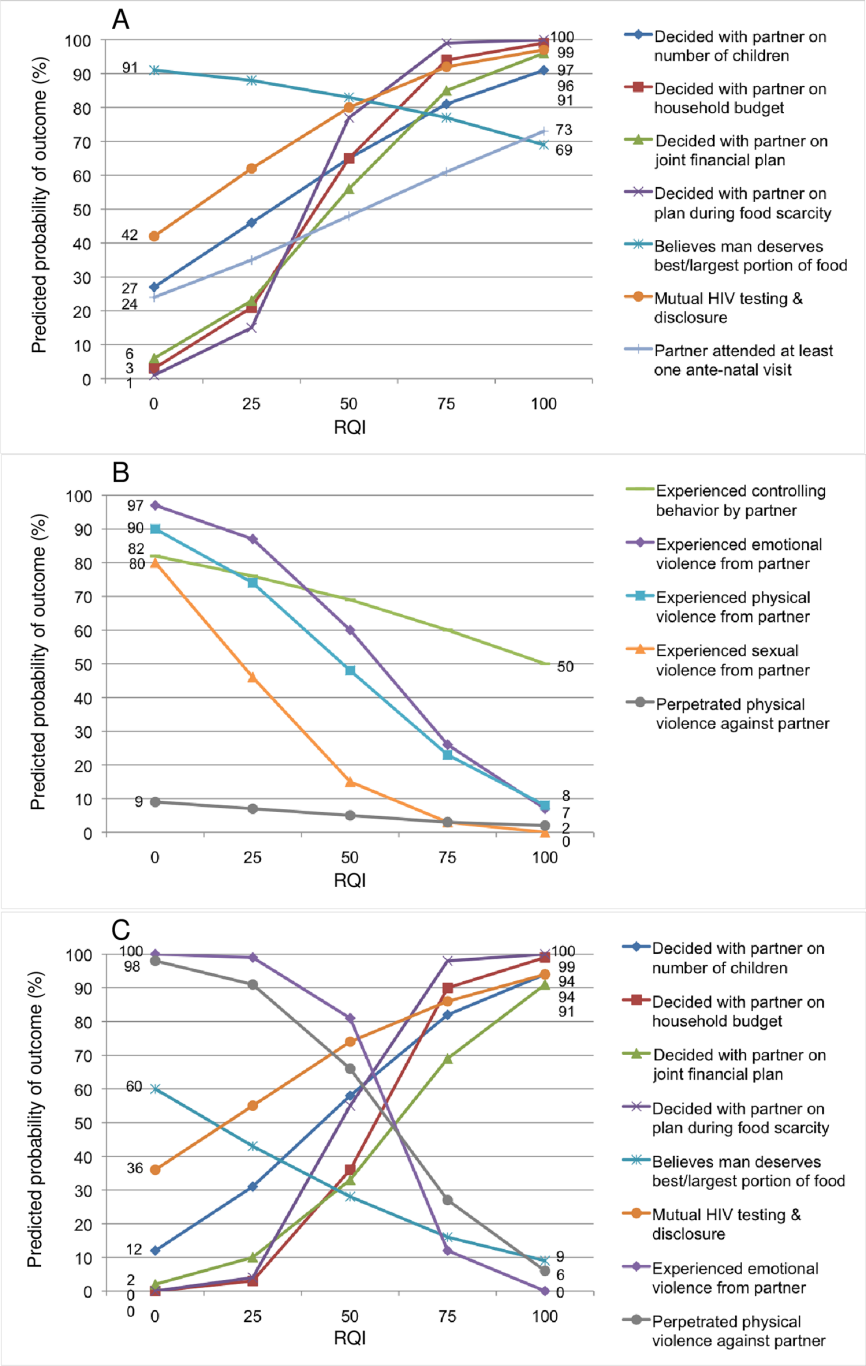
Predicted probabilities of outcomes by Relationship Quality Index (RQI). (A) Predicted probabilities of household cooperation, gender-equitable norms, and positive health behaviors at RQI quartiles, WOMEN. (B) Predicted probabilities of IPV at RQI quartiles, WOMEN. (C) Predicted probabilities of household cooperation, gender-equitable norms, positive health behaviors, and IPV at RQI quartiles, MEN.

For both women and men, we observed significant associations between the RQI (which included five domains of relationship quality) and the dependent variables measuring household cooperation, positive health behaviors, IPV, and gender-equitable norms. Fig 1 shows predicted probabilities of each outcome at RQI quartiles (scores of 0, 25, 50, 75 and 100). For example, a predicted 91% of women with an RQI of 0 would believe that the male partner deserved the best or largest portion of food at mealtimes, whereas a predicted 69% of women with an RQI of 100 would believe this (Fig 1A).

Table 4 shows predicted probabilities of the dependent variables (outcomes) for scores of 0 versus 100 (i.e. the endpoints of the lines in Fig 1) for the RQI and for each domain of relationship quality. For example, we predict that 27% of women with an RQI of 0 would report having decided with their partner how many children they want to have, whereas 91% of women with an RQI of 100 would report the same. The RQI was significantly associated (at p < .05) with all outcomes for women with three exceptions: gender-equitable norms, experiencing controlling behavior, and perpetrating physical violence. Seven of 12 associations were significant at p < .001. For men, the RQI was significantly associated (at p < .05) with all outcomes with two exceptions: gender-equitable norms and reporting mutual HIV testing and disclosure. Five of 8 associations were significant at p < .001. The domains of relationship quality showed multiple significant correlations with outcomes, which will be further explored below.

**Table 4:** Association of outcomes to relationship quality domains, final measurement model.

	Relationship Quality Index	Intimacy	Partner support	Sexual satisfaction	Positive communication	Joint decision-making
**Women (n = 203)**						
	% | %	% | %	% | %	% | %	% | %	% | %
*Decided with partner on*						
Number of children	27 | 91***	41| 84*	55 | 86*	29 | 87**	30 | 88*	74 | 85
Household budget	3 | 99***	11 | 96***	32 | 98***	13 | 96***	41 | 94	78 | 97*
Joint financial plan	6 | 96***	10 | 90***	20 | 94***	24 | 88**	22 | 91**	74 | 88
Plan during food scarcity	1 |100***	7 | 99***	18 |100***	24 | 98***	21 | 99*	85 |100*
*Health behaviors*						
Mutual HIV testing & disclosure	42 | 97**	39 | 95**	76 | 93	64 | 93	28 | 97**	86 | 93
Partner attended at least one ante-natal visit	24 | 73*	17 | 67*	38 | 67*	21 | 68*	37 | 66	60 | 60
*Gender norms*						
Man deserves best/ largest portion of food	91 | 69	67 | 78	86 | 73	88 | 74	98 | 64	80 | 72
*Experience of IPV*						
Controlling behavior	82 | 50	80 | 57	71 | 56	72 | 58	94 | 46*	63 | 58
Emotional violence	97 | 7***	97 | 17***	78 | 16***	82 | 21**	99 | 7***	36 | 24
Physical violence	90 | 8***	89 | 16***	66 | 14***	77 | 18**	97 | 8***	29 | 22
Sexual violence	80 | 0***	48 | 3**	29 | 2**	84 | 1***	93 | 1**	8 | 3
Perpetrated physical violence	9 | 2	35 | 2	2 | 4	3 | 4	4 | 3	4 | 3
**Men (n = 198)**						
	% | %	% | %	% | %	% | %	% | %	% | %
*Decided with partner on*						
Number of children	12 | 94**	3 | 91***	68 | 83	23 | 87*	9 | 91**	76 | 84
Household budget	0 | 99***	17 | 92**	15 | 97***	32 | 91*	8 | 94**	61 | 99***
Joint financial plan	2 | 91***	15 | 75*	5 | 88***	37 | 71	5 | 82**	60 | 75
Plan during food scarcity	0 |100***	13 | 98**	38 | 99**	65 | 97	1 |100***	87 | 99*
*Health behaviors*						
Mutual HIV testing & disclosure	36 | 94	43 | 90	62 | 91	23 | 92**	33 | 93	87 | 86
*Gender norms*						
Man deserves best/ largest portion of food	60 | 9	51 | 13	47 | 11*	53 | 12	72 | 10*	17 | 17
*Experience of IPV*						
Emotional violence	100 | 0***	100 | 5***	80 | 7***	90 | 10***	100 | 3***	35 | 6**
Perpetrated physical violence	98 | 6***	80 | 21*	75 | 16**	68 | 24	89 | 16**	43 | 14**

*Note*: Predicted probabilities (in %) of reporting each outcome for the lowest versus highest possible score for each relationship quality scale, or in the case of decision-making, for 0% versus 100% of decisions made jointly by both partners. All analyses adjusted for age, education, marital status, number of children in household, age difference between partners, and partnership duration.

* p < .05

** p < .01

*** p < .001

While we have reported thresholds of significance as liberal at alpha = 0.05, we urge caution in interpreting these results. Due to the large numbers of comparisons being made, we anticipate that some weak associations might be spurious. Further research will be required to build upon and confirm these results.

#### Household cooperation

For women, the relationship quality domains of increased intimacy, partner support, and sexual satisfaction were strongly and positively associated with household cooperation (p < .001 for 8 of 12 associations, p < .01 for 2 associations, and p < .05 for 2 associations). The weakest associations were seen with having decided with partner how many children to have, and the strongest associations were seen with deciding with partner how to manage the household budget, having a joint financial plan to which both contribute incomes, and having talked about what to do at times when there wasn’t enough food in the household. Somewhat surprisingly, the relationship quality domain of joint decision-making was only weakly associated with the outcome of household cooperation (2 of 4 associations significant at p < .05). The RQI encompassing all five relationship quality domains was a much stronger predictor of household cooperation, with associations between the RQI and all four household cooperation outcomes significant at p < .001. Finally, 3 of 4 associations between positive communication and household cooperation were significant at p < .05.

For men, the associations between household cooperation and increased intimacy, partner support and sexual satisfaction were somewhat weaker than for women (p < .001 for 3 of 12 associations, p < .01 for 3 associations, p < .05 for 3 associations, and 3 associations non-significant). The strongest associations were seen with deciding with partner how to manage the household budget (all associations significant), and weaker associations were seen with other variables (only 2 of 3 associations significant). Joint decision-making was more strongly associated with deciding with partner how to manage household budget for men than it was for women (p < .001 for men versus p < .05 for women). As with women, household cooperation was unexpectedly more strongly predicted by the RQI (all 4 associations significant at p < .01) than by reporting joint decision-making (2 of 4 associations significant at p < .05). Positive communication was more strongly associated with household cooperation for men than it was for women (p < .001 for 3 of 4 associations, p < .01 for 1 association).

#### Health behaviors

Significant associations were seen between relationship quality and both partners having been tested for HIV and mutually disclosed (according to participant’s report), for both men and women. Women who reported the lowest level of intimacy had a predicted probability of mutual HIV testing and disclosure of 39%, whereas for women who reported the highest level of intimacy the predicted probability was 95% (difference significant at p < .01). Similarly, women with the lowest versus highest levels of positive communication had predicted probabilities of mutual HIV testing and disclosure of 28% versus 97% (p < .01). For men, only sexual satisfaction was significantly associated with reporting mutual HIV testing and disclosure, with predicted probabilities of 23% and 92% for men with the lowest and highest levels of sexual satisfaction (p < .01). For women, weak associations were seen between reporting that a partner had attended at least one antenatal visit and intimacy, partner support, and sexual satisfaction (p < .05 for all).

#### IPV

Strong and consistent associations were seen between relationship quality and IPV, for both men and women. Women who reported higher intimacy, partner support, sexual satisfaction, and positive communication were significantly less likely to report experiencing emotional, physical, and sexual violence from their partners (p < .001 for 7 of 12 associations, p < .01 for 5 of 12 associations). No significant associations were seen between IPV and joint decision-making. Controlling behavior was prevalent among women at all levels of relationship quality, and only one weak association was seen, with women who reported more positive communication being less likely to report controlling behavior (p < .05). For men, lower relationship quality across the five domains was consistently associated with reporting emotional violence by partner (p < .001 for 4 of 5 associations, p < .01 for 1 association), and also with perpetrating violence against a partner (p < .01 for 2 of 5 associations, p < .05 for 2 associations). The only domain of relationship quality that was not associated with men perpetrating physical violence was sexual satisfaction.

#### Gender-equitable norms

Three-quarters of women believed that the male partner deserved the best or largest portion of food at mealtimes, and no domain of relationship quality was significant associated with this belief. Men who reported more partner support and more positive communication were less likely to believe they deserved the best or largest portion of food at mealtimes (p < .05 for both associations).

#### Strength of association by relationship quality domains

Finally, we consider which domains of relationship quality were most consistently and significantly associated with household cooperation, positive health behaviors, IPV, and gender-equitable norms. Intimacy, partner support, and positive communication were significantly associated with a majority of outcomes for both men and women (at least 8 of 11 associations for women, and at least 6 of 8 associations for men). Sexual satisfaction was significantly associated with a majority of outcomes for women (8 of 11), but only half of outcomes for men (4 of 8). Joint decision-making was most weakly associated with outcomes of interest, showing significant associations for only 2 of 11 outcomes for women, and 4 of 8 for men.

### Couple-level analysis

Notably, the number of men reporting ever having perpetrated physical violence against their partners (56) was nearly identical to the number of women who reported having ever experienced physical violence from partners (57). The women and men reporting violence were not necessarily reporting about the same relationships, as more than half of the sample did not have their partners included in the study. However, these similar rates suggest that women and men were reporting on violence similarly, and that it is unlikely that either gender was affected by a significant under- or over-report bias relative to the other gender. A further analysis of couples who were both interviewed in the study found that a majority agreed regarding whether physical violence had occurred in their relationship. Of the 89 couples in the study, 10 (11%) agreed that the man had perpetrated physical violence against the woman, while 54 (61%) agreed that the man had not perpetrated physical violence against the woman. Slightly over a quarter of couples disagreed (28%), with 11 women saying that the man had perpetrated violence when the man said he had not, and 14 men saying they had perpetrated violence when the woman said he had not.

In order to investigate whether or not knowing that one’s partner was also being interviewed biased responses, the responses of women and men whose partners had not been interviewed in the survey were compared to the responses of women and men whose partners had been interviewed in the survey. For most items and scales, responses did not vary significantly between the two groups. There were several exceptions. Women whose partners were not interviewed reported lower scores on the final intimacy scale (mean score of 83 versus 88, p < .05), and also on the final partner support scale (mean score of 69 versus 77, p < .05). Men whose partners were not interviewed similarly reported less gender-equitable attitudes on the gender roles scale (mean score of 38 versus 47, p < .05). Dependent and demographic variables did not differ significantly depending on whether the partner was or was not interviewed, for either men or women (see supporting information file).

Of the 89 couples included in the study, 70 (79%) agreed that they had both received an HIV test and disclosed their results, while 5 (6%) agreed that they had not both received an HIV test and disclosed results. For a further 3 couples (3%), the man reported that they had both tested and disclosed while the woman said they had not, while for 11 couples (12%), the women reported that they had both tested and disclosed while the man said they had not.

## Discussion

This study provided a wealth of data about couple functionality in a rural Malawian population and regarding the impact of various aspects of couple relationships on behaviors and attitudes which support key health and development goals. Furthermore, this validation study was successful in its goal of producing a tool to assess couple relationship quality that is short enough to be easily utilized in various program settings, and without a loss of reliability or validity. The RQI was shown to be valid for both women and men, and married and cohabiting individuals. The RQI showed significant predictive validity, for all groups surveyed, on a number of important behaviors. This stability across groups is encouraging and warrants replicating this study across other populations and contexts to determine the RQI’s validity and robustness across various settings. An analysis of couple-level data revealed that couples’ responses were not significantly affected by knowing that their partner was also being interviewed, which also supports the validity of the tool.

While women and men generally reported quite high relationship quality, the RQI was successful in distinguishing between various levels of relationship quality, and in showing that these differences can predict key behaviors such as household decision-making, HIV testing, and IPV. The domains of intimacy, partner support, and positive communication were most strongly associated with the behaviors of interest. These associations are not surprising; we would expect that couples with relationships characterized by these positive qualities would be more likely to cooperate and share decision-making on household matters such as finances and budgeting, practice healthy behaviors as a couple, and have low levels of IPV. Yet this study makes an important contribution by documenting the existence of such linkages between relationship quality and behaviors of interest to health and development professionals, and by demonstrating that relationship quality can be reliably measured with a simple tool.

The analysis did yield some more surprising findings, such as the high prevalence of violence for couples with all levels of relationship quality. Women in this study reported similar levels of violence compared to ever-married southern Malawian women aged 15–49 in the 2010 Malawi DHS [27]. In the DHS, 23% of women reported emotional violence, 22% reported physical violence, and 15% reported sexual violence. The comparable proportions among women in this study were 32%, 28%, and 5%. In the DHS, 65% of women reported some form of controlling behavior by a husband or partner, while in this study 61% of women did so. In this study, women with poorer quality relationships reported greater levels of violence, whereas no significant correlation was seen between reporting controlling behavior and reported relationship quality. More than half of women reported some form of controlling behavior by partners, suggesting a high level of social acceptability of such behaviors. In addition, approximately 1 in 3 women and 1 in 5 men reported emotional violence from a partner, such as public humiliation, threats, or insults. Such behavior showed a strong negative association with nearly all domains of relationship quality for both women and men.

This study also makes an important contribution by demonstrating the importance of sexual satisfaction as a domain of relationship quality, with associations to positive behaviors being particularly strong for women. While research from high-income countries shows strong linkages between sexual satisfaction and overall relationship satisfaction [28], most research in African contexts has focused on sexual relations from a problematic perspective, as vectors of violence or disease, rather than exploring the positive functions of sex within relationships [29]. Our study suggests the need for further research to elucidate how sexual satisfaction contributes to overall relationship quality and satisfaction, and how private practices of sexual intimacy may strengthen or weaken a couple’s relationship in ways that impact the public manifestations of couple and household functioning.

We assessed both joint and sole decision-making (according to self-report), and found that while joint decision-making was linked to a number of positive outcomes, sole decision-making was linked to a number of negative outcomes for women and men. This finding highlights the importance of couple communication and cooperation, rather than power being wielded by one partner alone. This study supports the view that it is not women’s empowerment *per se*, but rather cooperation between partners that is positively associated with desirable outcomes. Various studies have provided evidence of the benefits of joint decision-making between husband and wife in decisions about women’s health care, compared to men making decisions alone [30–33]. Other research has also supported the view that women’s autonomous decision-making may be sub-optimal compared to joint decision-making. In Nepal, women’s autonomy (control over household decisions and freedom of movement) was negatively associated with some measures of men’s involvement in their wives’ maternal healthcare, while intra-spousal communication was positively associated with men’s involvement [34]. Similarly, among South African couples female power was found to be negatively associated with relationship intimacy, while shared power was positively associated with intimacy, trust, mutually constructive communication, and lack of conflict [10].

This study found high rates of couple HIV testing, with 90% of women, 85% of men, and 88% of all respondents reported mutual HIV testing and disclosure. Of the 89 couples included in the present study, 70 (79%) agreed that they had both received an HIV test and disclosed their results. Rates of HIV testing were similarly high in a previous study in rural southern Malawi which found that in 87% of couples both partners reported that they had been tested for HIV and disclosed to their partners [35].

Relationship factors including decision-making and communication have long been identified as critical determinants of whether men and women seek HIV testing and disclose results to partners, as well as whether women experience negative reactions to disclosure such as violence [5]. Previous research in rural Malawi found that women and men who reported higher relationship unity (defined as discussing important matters, displaying care, and offering assistance when needed) were less likely to test for HIV [36]. Disclosure of HIV status to a sexual partner has been associated with a “smooth relationship” in Ethiopia [38], while fear of conflict with a partner and lack of relationship stability were associated with non-disclosure among pregnant women in Tanzania [39]. Among individuals in the United Kingdom who had disclosed their HIV-positive status to a partner, higher relationship quality predicted positive outcomes such as perceived psychological safety and emotional closeness within the relationship [37]. The authors concluded that relationship quality might act as a “risk or a resilience factor in the disclosure process,” and suggested that an assessment of relationship quality might be used to screen for particular vulnerability in the disclosure process [37].

Qualitative research has further illuminated the relationship dynamics which cause individuals to disclose HIV status or couples to seek couple HIV counseling and testing (CHCT), with relationship stability and trust emerging as important factors across multiple studies. In KwaZulu-Natal, South Africa, HIV-positive women reported during in-depth interviews that their partners were generally supportive when they disclosed HIV infection, but that the stability of a partnership was a critical factor in whether they disclosed [40]. The level of trust in a relationship has been identified as an important determinant of HIV disclosure among pregnant couples in South Africa [41] and couples in Uganda [42]. A qualitative study in Uganda found that men who reported marriages characterized by love, trust, and understanding were willing to seek CHCT, while men who felt their relationships lacked stability and trust were reluctant to accompany their partners to prevention of mother-to-child transmission (PMTCT) services because they feared being forced to undergo CHCT [43]. Similarly, Kenyan couples with “relationship-centered motivations” were more likely to test for HIV and disclose, whereas “self-centered motivations” were associated with mistrust [44]. Women and men in Malawi reported that HIV testing after marriage was an “unusual event” which signified possible infidelity and might incur a loss of trust [45].

The RQI is one of the first such tools to be developed for assessing couple relationship quality and functioning across multiple domains for use in LMIC. While the RQI was developed based on data from a population-based sample and validity was found to be good according to multiple criteria, the generalizability of these findings to other populations is as yet unknown. In addition, the large number of correlations examined may mean that some are spurious, and thus some domains of relationship quality may not be related to health and development indicators as theorized in this study. Further work is needed to build upon and confirm these findings. CRS is currently carrying out validation studies to assess the performance of the RQI in other African populations, and to further test the associations between domains of relationship quality and key health and development outcomes.

## Supporting information

S1 Table Comparison of participants whose partners were and were not interviewed for survey. (DOCX)
