# Supplementary material for: Measuring couple relationship quality in a rural African population: Validation of a Couple Functionality Assessment Tool in Malawi
Source: PLoS One. 2017 Nov 30;12(11):e0188561. doi: 10.1371/journal.pone.0188561 (PMC5708731; doi:10.1371/journal.pone.0188561)
Supplement: S1 Table — (DOCX) [file pone.0188561.s001.docx]

**S1 Table. Comparison of participants whose partners were and were not interviewed for survey.**

|  | **Women** | | | |  | **Men** | | | |
| --- | --- | --- | --- | --- | --- | --- | --- | --- | --- |
|  | Partner interviewed  (n = 89) | Partner not interviewed  (n = 114) |  |  |  | Partner interviewed  (n = 89) | Partner not interviewed  (n = 109) |  |  |
| **Relationship quality domains (independent variables)** | | | | | | | | | |
|  | mean (SD) | mean (SD) | p-value | power |  | mean (SD) | mean (SD) | p-value | power^†^ |
| Intimacy^†^ | 87.9 (15.2) | 82.6 (20.4) | 0.042* | 56% |  | 86.9 (11.5) | 87.3 (13.4) | 0.832 | 6% |
| Partner support^†^ | 77.0 (23.0) | 69.1 (27.9) | 0.032* | 60% |  | 75.1 (19.9) | 77.2 (19.3) | 0.439 | 12% |
| Sexual satisfaction^†^ | 84.3 (15.9) | 81.4 (17.7) | 0.225 | 23% |  | 87.0 (11.8) | 85.9 (14.7) | 0.547 | 9% |
| Positive communication^†^ | 73.7 (21.9) | 69.3 (23.1) | 0.174 | 28% |  | 81.8 (18.3) | 77.7 (19.1) | 0.124 | 34% |
| Joint decision-making^†^ | 40.2 (33.5) | 42.1 (31.6) | 0.686 | 7% |  | 49.7 (35.2) | 45.4 (33.4) | 0.377 | 14% |
| **Outcomes (dependent variables)** | | | | | | | | | |
|  | mean (SD) | mean (SD) | p-value | power |  | mean (SD) | mean (SD) | p-value | power |
| Number of children^‡^ | 3.94 (1.25) | 3.76 (1.23) | 0.303 | 18% |  | 4.12 (1.19) | 3.83 (1.49) | 0.127 | 35% |
| Household budget^‡^ | 4.38 (0.90) | 4.15 (1.09) | 0.105 | 39% |  | 3.87 (1.15) | 3.99 (1.17) | 0.449 | 12% |
| Joint financial plan^‡^ | 4.09 (1.11) | 3.92 (1.25) | 0.318 | 17% |  | 3.42 (1.48) | 3.37 (1.43) | 0.814 | 6% |
| Plan during food scarcity^‡^ | 4.43 (0.71) | 4.34 (0.86) | 0.452 | 12% |  | 4.46 (0.80) | 4.27 (0.91) | 0.115 | 36% |
| Man deserves best/ largest portion of food^‡^ | 3.92 (1.46) | 4.00 (1.26) | 0.681 | 7% |  | 2.00 (1.26) | 2.06 (1.30) | 0.765 | 6% |
|  | % (SD) | % (SD) | p-value | power |  | % (SD) | % (SD) | p-value | power |
| Attended 4 or more ante-natal visits at last/current pregnancy^‡^ | 48.2 (50.3) | 47.7 (50.2) | 0.946 | 5% |  |  |  |  |  |
| Partner attended at least one ante-natal visit at last/current pregnancy^‡^ | 65.1 (48.0) | 55.0 (50.0) | 0.156 | 30% |  |  |  |  |  |
| Both partners have been tested for HIV and mutually shared status | 92.0 (27.2) | 90.3 (29.8) | 0.661 | 7% |  | 82.0 (38.6) | 88.8 (31.7) | 0.178 | 26% |
| Controlling behavior by partner | 57.3 (49.7) | 62.3 (48.7) | 0.472 | 11% |  |  |  |  |  |
| Emotional violence by partner, ever | 32.6 (47.1) | 31.6 (46.7) | 0.879 | 5% |  | 20.2 (40.4) | 17.4 (38.1) | 0.616 | 8% |
| Physical violence by partner, ever | 23.6 (42.7) | 31.6 (46.7) | 0.209 | 25% |  |  |  |  |  |
| Sexual violence by partner, ever | 7.9 (27.1) | 3.5 (18.5) | 0.174 | 26% |  |  |  |  |  |
| Perpetrated physical violence against partner, ever | 3.4 (18.1) | 2.6 (16.1) | 0.758 | 6% |  | 27.0 (44.6) | 29.4 (45.8) | 0.710 | 7% |

*Note:* Power to detect the difference if the exact size of the estimated difference were reflective of the broader difference in these groups. For example, for the first item (intimacy score for women), in 56% of trials in which there was a true difference of 0.05 points (on a scale of 0 to 1) in these two groups of women, we would expect to detect the difference as significant.

* = p < 0.05.

^†^ Standardized to 0 to 100 scale, with higher scores denoting a higher-quality or more gender-equitable relationship. For decision-making, proportion of total household decisions made jointly with partner according to respondent’s report, on scale of 0 to 100.

^‡^ Items ranked on the following scale: 1 = Strongly disagree, 2 = Disagree, 3 = Neutral, 4 = Agree, 5 = Strongly agree.
